# Barriers and facilitators to telemedicine contraception among patients that speak Spanish: a qualitative study

**DOI:** 10.1016/j.xagr.2024.100428

**Published:** 2024-12-04

**Authors:** Marielle E. Meurice, Gennifer Kully, Sarah Averbach, Antoinette Marengo, Jesse Nodora, Maricela Cervantes, Sheila K. Mody

**Affiliations:** a Division of Complex Family Planning, Department of Obstetrics Gynecology and Reproductive Sciences, University of California San Diego, La Jolla, CA (Meurice, Kully, Averbach and Mody); b Planned Parenthood of the Pacific Southwest, San Diego, CA (Marengo); c Radiation Medicine and Applied Sciences, Moores Cancer Center, University of California San Diego, La Jolla, CA (Nodora); d California Latinas for Reproductive Justice, Los Angeles, CA (Cervantes)

**Keywords:** contraception counseling, Latine, telehealth, telemedicine, telecontraception

## Abstract

**Background:**

Telemedicine contraception services have increased since the COVID-19 pandemic. There may be unique equity implications and language barriers for patients who speak Spanish.

**Objective:**

To identify the barriers and facilitators of telemedicine for contraception care among patients who speak Spanish using a community-based participatory research approach.

**Study Design:**

The study was designed and conducted in consultation with a community advisory board. We interviewed 20 patients after telemedicine and in-person contraception visits conducted in Spanish at Planned Parenthood of the Pacific Southwest in Southern California between April 2022 and May 2023. Telemedicine visits were conducted by audio only. Two coders analyzed the data using thematic analysis.

**Results:**

The average age of the participants was 32.5 years old (range 19–45). Most participants had some college education (13/20, 65.0%) and public insurance (18/20, 90.0%). Most chose a short-acting contraceptive method (11/20, 55.0%). Five key themes were identified. (1) Participants reported less comfort with video technology and a preference to not be seen during the appointment, therefore preferring audio-only for telemedicine visits. (2) Participants did not report difficulty with Spanish interpreters using telemedicine. (3) Telemedicine has conveniences related to time, work, childcare, and transportation but may have inconveniences related to method receipt. (4) Preference for physical exam and preventative care and familiarity with the in-clinic model motivated people who sought in-person care rather than technology barriers with telemedicine. (5) There is trust in the privacy and confidentiality of the visits, but privacy at home for the individual may impact choice for in-person care.

**Conclusion:**

Among patients who speak Spanish, telemedicine contraception care was acceptable and had many conveniences. Many patients who speak Spanish preferred audio-only for telemedicine contraception visits. Use of interpreters and technology were not perceived barriers to care.

**AJOG Global Reports at a Glance**

**Why was this study conducted?**

Telemedicine contraception services have increased and may have equity implications related to technology access and language barriers.
This study identifies the barriers and facilitators of telemedicine for contraception care among patients who speak Spanish using a community-based participatory research approach.

**Key findings**

Among patients who speak Spanish, telemedicine contraception care was acceptable with many conveniences.
Audio-only technology was preferred by most.
Interpreters were not perceived as barriers to care.

**What does this study add to what is known?**

People can determine if telemedicine contraception meets their needs and preferences.
Providing audio options for telemedicine contraception with interpreter services supports equity and patient preference.

## Introduction

Telemedicine use increased during the COVID-19 pandemic.^1^ While telemedicine has benefits, it has the potential to marginalize populations, increase inequities, and limit access due to language barriers.^2^^,^^3^ During the early COVID-19 pandemic, increased age, non-English language, female gender, Latine ethnicity, and lower income were associated with increased phone visits compared with video visits for primary and ambulatory care.^4^ This may suggest decreased access to technology and potentially disparate care. Telemedicine benefits include decreased need for transportation, shorter time needed to allocate to the appointment, and scheduling flexibility.^5^

Before the COVID-19 pandemic, telemedicine use was increasing and showed promise for family planning.^6^ Clinicians and patients reported high satisfaction with telemedicine contraception during the pandemic in New York City.^7^^,^^8^ An exploratory study found no meaningful difference in self-reported quality of contraception counseling between in-person and telemedicine visits.^9^

Patients who had appointments in Spanish have reported unique preferences and experiences with contraception counseling, such as a preference for a language-concordant clinician,^10^ more difficulty communicating concerns, and feeling rushed.^11^ This may create unique challenges for telemedicine contraception. A recently published exploratory study of telemedicine contraception visits showed people who spoke Spanish rated the quality of their visits lower compared with English speakers.^12^

The objective of the study is to identify barriers and facilitators for using telemedicine for contraception care among patients who speak Spanish in Southern California. We hypothesize that telemedicine patients may encounter more language and technology barriers than those who had in-person visits, but there also might be improved access through convenience and decreased need for transportation and childcare.

## Materials and methods

**Figure 1:**
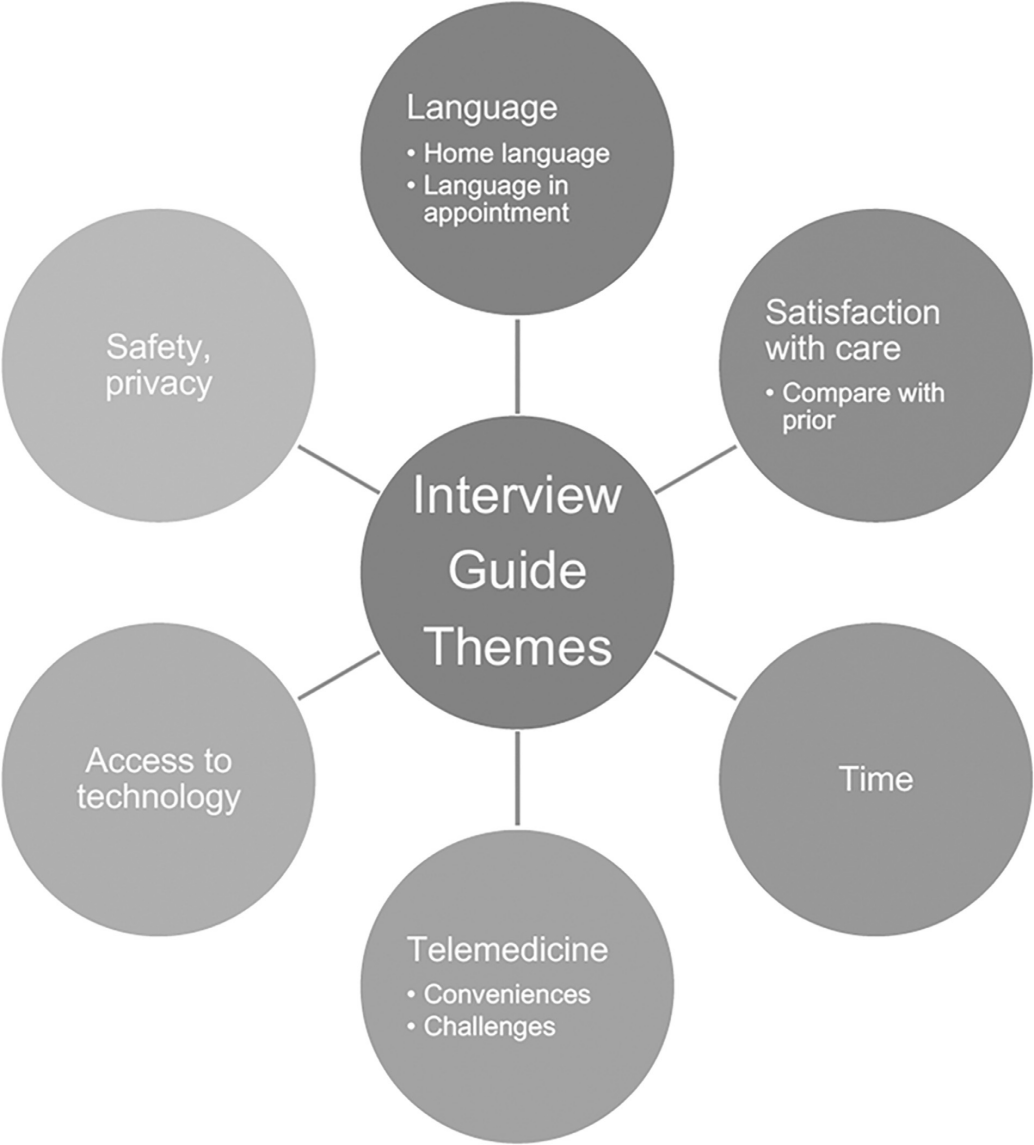
Interview guide probes exploring barriers and facilitators to telemedicine contraception among patients that speak Spanish at Planned Parenthood of the Pacific Southwest 2022–2023

The University of California, San Diego IRB approved this study. We utilized community-based participatory research methodologies in study design and data interpretation. We partnered with local Latine community organizations and created a 6-member community advisory board (CAB). Organizations and individuals were found by internet-based research and/or word of mouth. Our CAB included advocates from California Latinas for Reproductive Justice, Latinos in Clinical Research, the Wound Clinic, and a clinician and patient access specialist from Planned Parenthood of the Pacific Southwest (PPPSW). We created anticipated interview themes (Figure 1) and a guide (Appendix A) and reviewed the findings iteratively during data collection and the final data analysis.

Meurice. Barriers and facilitators to telemedicine contraception among patients that speak Spanish: A qualitative study. AJOG Glob Rep 2024.

The novel, semi-structured interview guide (Appendix A) focused on consultation experience, language preferences and interpreter experience, reason for choosing visit type, adequacy of time, opinion of telemedicine, barriers/facilitators to using telemedicine, and internet/device access.

We recruited from Planned Parenthood of the Pacific Southwest (PPPSW), which serves Riverside, Imperial, and San Diego counties. We included participants who had contraception counseling in-person or via telemedicine in Spanish and were 18-49 years old. Language preference was asked during intake and if Spanish was preferred, the visit was conducted in Spanish with an interpreter. All counseling was provided by advanced practice clinicians (APC) and used a shared decision-making model. Clinics were in El Cajon, Escondido, Imperial Valley, and San Diego. Telemedicine services were centralized for all counties. Spanish telemedicine visits were conducted only with audio-only due inability to include the interpretation service on a video call. The clinician called the interpreter, and the visit was done as a three-way call with the patient.

People who had a telemedicine contraception appointment were recruited at the end of the encounter. A pre-survey link was sent via text. People who had in-person contraception appointments were given a study flier and could access the pre-survey via text or QR code. We came to recruit after in-person appointments on two dates. The pre-survey included demographic information (age, education, insurance, and county of residence) and the validated Person-Centered Contraceptive Counseling scale (PCCC).^13^ The PCCC scale has 4 items and uses a 5-point Likert scale to measure the quality and person-centeredness of contraception counseling. The measure result is calculated as the percentage of people that give all the items top scores (all 5s on the Likert scale).^14^ After completing the form, we contacted individuals by phone or email and offered them an interview. We conducted our interviews by phone, Zoom, or in-person per patient preference irrespective of their visit type. We obtained verbal informed consent before the start of the interviews. We conducted all interviews in Spanish with GK, and they lasted approximately 30 minutes. The research coordinator (GK) conducted the interviews and is fluent in Spanish and certified interpreter, has a master's degree in research, and has prior experience conducting qualitative interviews. We compensated participants with gift certificates. The audio was recorded, professionally transcribed, and translated into English for analysis.

We performed thematic analysis^15^ using Atlas.ti version 23.1.1. Two researchers (MM, GK) reviewed the transcripts to develop themes and eventually a codebook, building upon the framework created by the CAB. Coders worked to code 3 transcripts together to ensure mutual understanding of the codes and refine the codebook as needed. Next, 5 transcripts were coded separately and high intercoder reliability was calculated (Krippendorff's alphas 0.852). Thereafter we coded independently for the remaining 12 transcripts. We resolved questions by consensus by the 2 researchers (MM, GK) and themes were grouped and summarized. There were 20 planned interviews and the authors felt that thematic saturation was reached after these interviews were completed.

## Results

Between April 2022 and May 2023, we completed 20 qualitative interviews – 10 after in-person appointments and 10 after telemedicine appointments. For the in-person cohort, 26 individuals started the presurvey. Of those, 7 did not qualify for the study due to age or appointment language. The remaining 9 either did not fully complete the pre-survey or did not respond to the interview invite. Therefore, 10/16 qualifying, interested individuals who had in-person visits were interviewed. Of the 10 in-person cohort interviews, 4 were conducted in clinic and the remaining 6 were done by phone. For the telemedicine cohort, 34 people opened the pre-survey. There was 1 individual who did not qualify for the study and 23 individuals who did not complete the pre-survey. Therefore, 10/10 interested, qualifying individuals who had telemedicine appointments were interviewed. All interviews for the telemedicine cohort were done by phone.

The average age of the participants was 32.5 years old (range 19–45). All participants identified as cis-gendered females. Most participants had at least some college education (13, 65.0%) and public insurance (18, 90.0%). All participants reported subjectively being satisfied with their care. The quality of care was similar between the in-person and telemedicine groups, and 9 (45.0%) reported PCCC top scores. The most common types of contraception were short-acting methods (11, 55.0%) (Table 1).

**Table 1:** Study participant characteristics exploring barriers and facilitators to telemedicine contraception among patients that speak Spanish at Planned Parenthood of the Pacific Southwest 2022-2023 (n = 20)

Participant characteristic	Telemedicine (n = 10)	In-person (n = 10)	Total (N = 20)
	n(%)		
Interview modality			
Phone	10 (100)	5 (50)	15 (75)
Zoom	0 (0)	1 (10)	1 (5)
In-person	0 (0)	4 (40)	4 (20)
Age - Mean (range)	32.7 (23–45)	32.4 (19–42)	32.5 (19–45)
Education			
Secondary school or less	5 (50.0)	1(10.0)	6 (30.0)
Some college	3 (30.0)	4 (40.0)	7 (35.0)
College	1 (10.0)	4 (40.0)	5 (25.0)
Graduate school	1 (10.0)	0 (0.0)	1 (5.0)
Prefer not to answer	0 (0.0)	1 (10.0)	1 (5.0)
Insurance			
FamilyPACT	5 (50.0)	2 (20.0)	7 (35.0)
Medi-Cal	4 (40.0)	7 (70.0)	11 (55.0)
Unknown	1 (10.0)	1 (10.0)	2 (10.0)
County of residence			
Imperial	1 (10.0)	5 (50.0)	6 (30.0)
Riverside	7 (70.0)	2 (20.0)	9 (45.0)
San Diego	2 (20.0)	3 (30.0)	5 (25.0)
Top Score PCCC^a^	4 (40.0)	5 (50.0)	9 (45.0)
Contraception choice^b^			
Pill/Patch/Ring	6 (60.0)	5 (50.0)	11 (55.0)
EC	1 (10.0)	0 (0.0)	1 (5.0)
Injection	1 (10.0)	1 (10.0)	2 (10.0)
LARC	2 (20.0)	3 (30.0)	5 (25.0)
Condoms	0 (0.0)	1 (10.0)	1 (5.0)
PFC	0 (0.0)	1 (10.0)	1 (5.0)
Undecided	1 (10.0)	0 (0.0)	1 (5.0)

FamilyPACT, Family Planning, Access, Care, Treatment program, a California program that provides free services to low-income residents. Medi-Cal, California's Medicaid program; PCCC, person-centered contraception counseling; EC, emergency contraception; LARC, long-acting reversible contraception; PFC, permanent female contraception.

a Participants who gave all 5′s to the person-centered contraception counseling (PCCC) measure which is a four question quality measure that measures person-centeredness of contraception counseling. Responses range from 1 (poor) to 5 (excellent)

b Could choose multiple, reflected in denominator.

All participants reported access to a phone, whereas about half of the participants in both groups had access to a tablet or computer. Only one telemedicine participant reported a minor issue with their connection, whereas more than half of the in-person participants had concerns regarding internet and/or phone connection when considering a theoretical telemedicine visit.

Five main themes were identified during the analysis.

Theme 1 Participants reported less comfort with video technology and a preference to not be seen during the appointment, preferring audio-only for telemedicine visits.

When asked about their preference for audio versus video-audio technology for the visit, the majority reported a preference for audio-only (14/20). For some participants, this preference was related to familiarity with phone technology and lack of comfort with video technology, with one individual reporting,

*“Because it's easier because I'm familiarized with that, with the basic technology. I don't know how to receive a video call, and I don't think it's very convenient.‬”*

*45-year-old from Riverside County who chose OCPs after telemedicine visit*

Other participants were less comfortable being seen as they may have their children nearby, were concerned about their appearance, or felt embarrassed. One individual described this hesitation to be seen and preference for audio-only,

*“It seems better for me, right, because I'm very shy. That's why because it's a little embarrassing.‬”*

*40-year-old from Imperial County who chose OCPs after telemedicine visit*

Two participants preferred video, one from each cohort. They both expressed a preference to be able to see their doctor,

*“Through a video phone call, I would feel more secure with speaking to the doctor in front of me. To have more visual contact with the doctor. It's a good option for me.”‬*

*27-year-old from San Diego County who chose injection after telemedicine visit*

*“[I prefer] video because as I mentioned earlier, they can see my movements, and it's easier with my hands.”* ‬‬‬‬‬‬‬‬‬‬‬‬‬‬‬‬‬‬‬‬‬‬‬‬‬‬‬‬‬‬‬‬‬‬‬‬‬‬‬‬‬‬‬‬‬‬‬‬‬‬‬‬‬‬‬‬‬‬‬‬‬‬‬‬‬‬‬‬‬‬‬‬‬‬‬‬‬‬‬‬‬‬‬‬‬‬‬‬‬‬‬‬

*‬30-year-old from San Diego County who chose OCPs after in-person visit‬*‬

Theme 2 Participants did not report difficulty with Spanish interpreters using telemedicine*.*

The majority (12, 60.0%) of the participants identified as monolingual Spanish speakers. One participant spoke Spanish as a second language and preferred a language from Guatemala, Q'anjob'al, and was the only participant who described any difficulties related to the use of an interpreter. One monolingual Spanish speaker responded,

*“I didn't have any difficulties, no. But at first, they did speak to me in English. However, they sought an interpreter for me right after, and I was able to finish the rest of my appointment.”*

*29-year-old from Riverside County who chose OCPs after telemedicine visit*

Seven participants identified as bilingual, all of whom preferred Spanish to discuss contraception care due to the nuances and seriousness of the topic. The in-person cohort was more likely to have a language-concordant clinician or medical assistant, but almost all used an interpreter at some point in their visit.

Theme 3 Telemedicine has conveniences related to time, work, childcare, and transportation but may have inconveniences related to method receipt.

When asked about benefits of telemedicine – either in theory for the in-person cohort or experienced in the telemedicine cohort – almost all participants felt there were many conveniences related to time (19/20), work (8/20), childcare (11/20), and transportation (13/20).

Summarizing their experience, one participant stated,

*“It was quicker and safer to do it because of my schedule, work, my children, and everything else…It was a secure phone call, confidential and I felt very safe with them.‬”*

*35-year-old from Riverside County who chose IUD removal after telemedicine appointment* ‬‬‬‬

This contrasts with potential inefficiencies described with the in-person model. One participant describes the inefficiency of her in-person visit,

“*I don't drive so my boyfriend had to give me a ride there. He took me there. He waited outside, and I went inside. I also had to check my work schedule. It's very flexible, but it would save me so much time, and it would've been much easier to do it on the phone.‬”*

*30-year-old from San Diego County who chose OCPs after in-person visit*

Childcare was reported as a potential convenience of telemedicine by most who had children (11/13). However, one participant reported that she still had someone watch their children during the appointment via telemedicine. Another participant described why she chose telemedicine,

*“It is sometimes difficult to find someone to leave my children with… this time I was given the option and chose to do it over the phone because I didn't have someone to take care of my children.‬”*

*27-year-old from Riverside County who chose the patch after telemedicine visit*

Some also reported that telemedicine may have conveniences related to work (8/20). People spoke about flexibility to take a call during work hours without having to physically leave.

*“Since I work then, it makes it better for me coming out of work. [It takes] a long time to go to the place where the office is at. So, it seems more comfortable to me to take a moment than going out for a longer amount of time.‬”*

*40-year-old from Imperial Valley who chose OCPs after telemedicine visit*

Most participants reported that telemedicine would be more convenient secondary to transportation concerns and its associated time and costs (13/20). Despite these conveniences, a few telemedicine participants reported difficulty with getting their prescriptions (2/10). One individual recounted the experience of trying to get OCPs and ended up changing her method to injection after a follow-up appointment as she was already in the clinic,

“*First I called [and] they would send the prescription to the CVS pharmacy near to where I live. When I went to pick up the pills, I had an incident when they asked for the code, I gave them the code they gave me, but they said that it wasn't the correct one. And that's why I called again, and they recommended that I visit the medical office so that they could evaluate me while I was there, and they could give me the birth control I selected.‬ […] Yes, the process and information were complicated.‬”*‬‬‬‬‬‬‬‬‬‬‬‬‬‬‬‬‬‬‬‬‬‬‬‬‬‬‬‬‬‬‬‬‬‬‬‬‬‬‬‬‬‬‬‬‬‬‬‬‬‬‬‬‬‬‬‬‬‬‬‬‬‬‬‬‬‬‬‬‬‬‬‬‬‬‬‬‬‬‬‬‬‬‬‬‬‬‬‬‬‬‬‬

*27-year-old from San Diego who chose Injection after telemedicine visit* ‬‬‬

Half of the telemedicine participants required a follow-up appointment or needed to pick up a prescription, whereas only two in-person participants required follow-up.

Theme 4 Preference for physical exam and preventative care and familiarity with the in-clinic model motivate people to seek in-person care rather than technology barriers with telemedicine.

Although more in-person than telemedicine participants voiced a concern regarding internet or phone connectivity, there was more focus on the preference to be seen and undergo physical exam and preventative care when asked about advantages of in-person visits. Additionally, many in-person patients used the walk-in system for their contraception appointment and found this convenient. Just under half of the in-person participants (4/10) voiced a preference for a physical exam and/or getting preventative care services such as sexually transmitted infection (STI) screening and cervical cancer screening while receiving contraceptive counseling. One person described this:

*”Maybe you have to get a urine or blood test, and then you will have to go in person. Because they're just giving you the information. Or maybe you need medication or something. You're going to have to leave the house either way.‬”* ‬‬‬‬‬‬‬‬‬‬‬‬‬‬‬‬‬‬‬‬‬‬‬‬‬‬‬‬‬‬‬‬‬‬‬‬‬‬‬‬‬‬‬‬‬‬‬‬‬‬‬‬‬‬‬‬‬‬‬‬‬‬‬‬‬‬‬‬‬‬‬‬‬‬‬‬‬‬‬‬‬‬‬‬‬‬‬‬

*32-year-old from San Diego County who chose OCPs after in-person visit* ‬‬‬‬‬‬‬‬‬‬‬

The desire for preventative care and physical exam wasn't discussed as a drawback in the telemedicine cohort. In fact, one voiced that talking on the phone decreased the fear of needing to undergo a physical exam.

Theme 5 There is trust in the privacy and confidentiality of the visits, but privacy at home for the individual may impact choice for in-person care.

‬‬‬‬‬‬‬All individuals reported feeling comfortable discussing family planning in general. However, telemedicine may have advantages in terms of making patients feel more at ease talking about sensitive topics. One individual thought that telemedicine might be even more comfortable, stating,‬‬‬‬‬‬‬‬‬‬‬‬‬‬‬‬‬‬‬‬‬‬‬‬‬‬‬‬‬‬‬‬‬‬‬‬‬‬‬‬‬‬‬‬‬‬‬‬‬‬‬‬‬‬‬‬‬‬‬‬‬‬‬‬‬‬‬‬‬‬‬‬‬‬‬‬‬‬‬‬‬‬‬‬‬‬‬‬

“*I think it's a comfortable thing to do because sometimes you feel better or more comfortable talking on the phone about those topics that can sometimes be a little bit painful to discuss with someone in person. So, I think it's a very good option.‬”*

*31-year-old from Riverside County who chose patch after in-person visit*

Privacy was not a primary concern for those who chose telemedicine, both in the sense of having a private place at home and the confidentiality of the visit through phone or video. However, a few participants (2/10) from the in-person cohort didn't feel they had a private space to talk in their homes and were concerned that their family might overhear something sensitive. Below a participant described trusting the system but that her own home might be a privacy concern,

“*When I have appointments online, before we start, they tell me that they're not recording anything. ‬“This is confidential, and it will not leave this space.” I don't have an issue with that. I think that the only privacy issues I would experience would be on my end in case anyone was to come into the space where I am.”*

*30-year-old from San Diego County who chose OCPs after in-person visit*

## Discussion

### Principal findings

This qualitative study provides insights into the experience and preferences regarding telemedicine contraception for individuals who speak Spanish. Contrary to our hypothesis, the experience with interpreters via telemedicine was neutral to positive and not identified as a barrier. Audio technology was strongly preferred by most. Technology barriers do exist, but preference for in-person care came more from a desire for physical exam and preventative services. The in-person cohort had a few concerns about internet connection, particularly in rural settings, as well as privacy related to telemedicine. Many participants felt there were conveniences for telemedicine however receipt of contraception frequently required additional time.

### Results

We do not yet understand if the quality of contraception care differs between telemedicine and in-person contraception care as quality appeared similar between both groups. Our findings that PCCC were similar between the two cohorts is consistent to a prior exploratory study.^9^

The inability to immediately provide a method through telemedicine could impact counseling. Additionally, the use of video could also lead to bias when seeing a patient's home or living situation. A prior study that focuses on clinician bias, found that differing appearances, race, and socioeconomic statuses changed whether a LARC was offered.^16^

Our community advisory board identified privacy as a central theme. A prior study of clinicians also identified this as a potential drawback of telemedicine contraception.^17^ A few of the in-person individuals listed privacy as a reason to seek in-person care instead of telemedicine. However, a few also voiced concern about privacy when seeking in-person care in a rural community. In the telemedicine cohort, there were no concerns regarding privacy/confidentiality, and some preferred the anonymity of the phone call.

### Clinical implications

Our findings regarding preference for audio-only technology, privacy concerns, role of the physical exam, and use of interpreters give clinicians insight into patient experiences and may be useful for their practices. This small, exploratory study inspires larger research questions that may help shape future policies and practice changes.

### Research implications

Further study of audio versus video visits may clarify the quality of these services. Additionally, a study of the perceived quality and person-centeredness of telemedicine contraception in Spanish versus English is merited.

### Strengths and limitations

Strengths of this study include recruitment from diverse settings. We focused on Latine persons, who are historically underrepresented in clinical research.^18^^,^^19^ Additionally, community-based participatory research methods strengthen the validity of our analyses and center patients’ voices and communities. Limitations include the small sample size, wide age range from 19 to 45 that may influence responses, and selection bias. Additionally, due to interpretation platform limitations, our participants had audio-only telemedicine visits. However, we queried preference for audio and video for a theoretical future visit to gather more information about the use of video for telemedicine contraception care. Finally, we only interviewed 10/16 individuals from the in-person cohort who showed interest in the study.

## Conclusions

This qualitative study that uses community-based participatory research methods attempts to understand and compare the experience of Spanish-speaking individuals seeking contraception care via telemedicine versus in-person and describes the barriers and facilitators for telemedicine care. Audio-only visits were preferred by most. There are many conveniences of telemedicine, but it's not ideal for everyone and people should be able to choose based on preferences and needs.

## CRediT authorship contribution statement

**Marielle E. Meurice:** Writing – review & editing, Writing – original draft, Validation, Project administration, Methodology, Investigation, Funding acquisition, Formal analysis, Data curation, Conceptualization. **Gennifer Kully:** Writing – review & editing, Writing – original draft, Validation, Project administration, Methodology, Investigation, Formal analysis, Conceptualization. **Sarah Averbach:** Writing – review & editing, Supervision, Methodology, Conceptualization. **Antoinette Marengo:** Writing – review & editing, Supervision, Methodology, Investigation, Conceptualization. **Jesse Nodora:** Writing – review & editing, Supervision, Methodology, Investigation, Conceptualization. **Maricela Cervantes:** Writing – review & editing, Validation, Methodology, Investigation, Formal analysis, Conceptualization. **Sheila K. Mody:** Writing – review & editing, Supervision, Resources, Project administration, Methodology, Investigation, Funding acquisition, Conceptualization.

## Declaration of competing interest

Sarah Averbach has served as a consultant for Bayer. Antoinette Marengo has served as a consultant for Organon, Daré, and Healthy Women, was on the speaker bureau for Bayer, and serves on the scientific advisory board for Afaxis pharmaceutical. Sheila Mody has served as a consultant for Bayer and Cadence Health Incorporated, and is an UptoDate author. The other authors have no conflict of interest.

## Appendix Supplementary materials

Image, application 1

## Footnotes

Supplementary material associated with this article can be found in the online version at doi:10.1016/j.xagr.2024.100428.
