# Supplementary material for: Barriers and facilitators to telemedicine contraception among patients that speak Spanish: a qualitative study
Source: AJOG Glob Rep. 2024 Dec 4;5(1):100428. doi: 10.1016/j.xagr.2024.100428 (PMC11762195; doi:10.1016/j.xagr.2024.100428)
Supplement: Supplementary file 1 [file mmc1.doc]

**Final Interview Guide (Spanish Version)**

Preguntas clave:

1. Por favor, cuéntame sobre su decisión de participar en este estudio.
2. ¿Cuál o cuales idiomas prefiere?¿ En este momento estamos hablando en su idioma preferido? ¿Cambia esta preferencia en diferentes contextos?

*Sondeo: en la casa, en el trabajo, en la clínica de salud, etc.*

1. Piense sobre su reciente cita en Planned Parenthood, cuénteme sobre su cita y la elección de anticonceptivos:
   1. ¿Por qué eligió que su cita fuera por medio de{telemedicina/en persona}?
   2. ¿Estuvo satisfech(a/o) con su atención? Por qué o por qué no.
   3. ¿Tuvo suficiente tiempo para hablar sobre sus necesidades o dudas en su cita? Por favor, explique por qué.
   4. ¿Tuvo dificultades con el idioma durante su cita? ¿ Su proveedor hablaba con fluidez en su idioma preferido? ¿Uso un interprete?
   5. ¿Hay algo que hubiera mejorado su experiencia?
   6. ¿Necesitó aglúna cita de seguimiento? Describa cualquier dificultad que encontró.

*Sondeo: tiene una cita agendada*

1. Hablemos del uso de la telemedicina (teléfono o video) utilizada para las consultas sobre métodos anticonceptivos o la planificación familiar.
   1. ¿Qué hace que la telemedicina sea fácil o cómoda para las consultas utilizadas para discutir métodos anticonceptivos?

*Sondeo: transporte, tiempo, motivo relacionado con el trabajo, cuidado de los niños, costos, acceso al centro.*

- 1. ¿Qué dificultades {encontró/podrían} encontrarse al utilizar la telemedicina?

*Sondeo: privacidad, idioma, dificultades tecnológicas, tiempo.*

- 1. ¿Le preocuparía hablar sobre su planificación familiar o salud reproductiva? ¿Tiene alguna preocupación acerca su privacidad o seguridad durante las consultas?
  2. Hábleme sobre su acceso al internet, computadoras y teléfonos inteligentes. ¿Qué aparato tecnológico prefiere que sea utilizado en una cita tele-médica para la planificación familiar? ¿Qué aplicaciones o plataformas encuentra fáciles de utilizar? Prefiere video o teléfono?

*Sondeo: WhatsApp, Zoom, etc.*

- 1. Hábleme sobre lo que necesitó organizar en su vida antes de su cita y si esto sería distinto con una cita por telemedicina. ¿Hay alguna hora del día o día de la semana que sea mejor para cita por telemedicina?

*Sondeo: transporte, tiempo, motivo relacionado con el trabajo, cuidado de los niños, horas de citas no tradicionales.*

Esas son todas las preguntas que tengo, ¿hay algo más que debería saber o que le gustaría decirme o comentarme?

Muchas gracias por su participación.

**Final Interview Guide (English version)**

Key Questions:

1. Please tell me about your decision to participate in this study.
2. Tell me about your preferred languages? Are we currently speaking in your preferred language? Does it change in different settings?

*Probe: at home, at work, etc*

1. Think about your recent appointment at Planned Parenthood, tell me a little about your appointment and contraception choice.
   1. Why did you choose {telemedicine/in person}?
   2. Were you satisfied with your care? Why or why not.
   3. Did you have enough time? Please explain.
   4. Were there any language difficulties during your appointment? Was your provider fluent? If not, did you use an interpreter?
   5. What, if anything, would have improved your experience?
   6. Did you need any follow-up care? Describe any challenges.
2. Let’s discuss the use of telemedicine (phone and video) for contraception visits.
   1. What makes telemedicine easy or convenient for contraception visits?

*Probe: transportation, time, work-related, childcare, costs, access to facility*

- 1. What challenges {were/would be} encountered for telemedicine?

*Probe: privacy, language, technological difficulties, time*

- 1. Would you have any concerns about talking about family planning or reproductive health? Do you have any privacy or safety concerns during visits?
  2. Tell me about your access to internet, computers, and smart phones. What would be best for you for a telemedicine contraception appointment? What apps or platforms are you comfortable using? Do you prefer video or telephone visit?

*Probe: WhatsApp, Zoom, etc.*

- 1. Tell me about anything you needed to arrange in your life for your {telemedicine/in-person) appointment and if this differs for telemedicine compared to in-person appointments. Is there a time or day of the week that is best for telemedicine?

*Probe: transportation, time, work-related, childcare, non-traditional appt times*

Those are all the questions I have, was there anything else I should know or that you'd like to tell me?

Thank you so much for your participation.
